# Perception of Social Support and Cognitive Performance in Older Adults With Depression

**DOI:** 10.1001/jamanetworkopen.2023.3978

**Published:** 2023-03-21

**Authors:** Raura Doreste-Mendez, Lauren E. Oberlin, Irena Ilieva, Serena Z. Chen, Faith M. Gunning, Nili Solomonov

**Affiliations:** 1 Weill Cornell Institute of Geriatric Psychiatry, Weill Cornell Medical College, White Plains, New York

## Abstract

This cross-sectional study assesses the association between perceived social support and cognitive performance in older adults with depression.

## Introduction

Approximately half of older adults with major depression experience comorbid cognitive difficulties.^1^ Executive dysfunction is associated with poor antidepressant medication response and often persists after remission of a depressive episode.^2^ Another risk factor associated with poor depression outcomes in older adults is poor social support, which is associated with chronicity and limited treatment response. Perceived social support has been reported as the domain of social support most strongly associated with depression severity and persistence.^3^ Executive processes, including initiation, planning, and persistence, are vital for social functioning, and studies in healthy older adults have observed associations between social support and executive functions.^4,5^ However, the association between these 2 important risk factors in late-life depression has not been well studied. In this study, we hypothesized that higher perceived social support would be associated with better executive function.

## Methods

This cross-sectional study was approved by the Weill Cornell Medicine institutional review board. All participants provided written informed consent. The study is presented following the Strengthening the Reporting of Observational Studies in Epidemiology (STROBE) reporting guideline.

We analyzed pretreatment data collected in an escitalopram treatment trial. Our sample included older adults (age 59-85 years) who met *Diagnostic and Statistical Manual of Mental Disorders* (Fourth Edition) (*DSM-IV*) criteria for major depressive disorder without psychotic features or dementia. Further details are provided in the eMethods in Supplement 1. We assessed global cognitive functioning (Dementia Rating Scale), depression severity (Montgomery-Åsberg Depression Rating Scale [MADRS]), and perceived social support (Duke Social Support Index Scale). Neuropsychological assessment included tests of semantic verbal fluency (Animal Naming Test), phonemic verbal fluency (Controlled Oral Word Association Test [FAS]), cognitive inhibition (Stroop Interference), set-shifting (Trail Making Test part B − A [TMT B-A]), memory (Hopkins Verbal Learning Test delayed recall), processing speed (TMT part A [TMT-A]), and self-reported executive function (Frontal Systems Behavior Scale–Executive Dysfunction subscale).

We conducted multiple linear regression analyses to examine the association between perceived social support and cognitive performance on measures of inhibitory control, set-shifting, phonemic fluency, semantic fluency, memory, processing speed, and self-reported executive function. All models were adjusted for age, sex, education, and baseline MADRS score. We used a Bonferroni correction for 7 two-tailed comparisons (*P* < .007). We tested reverse association (ie, cognitive functioning associated with social support) for any significant models. Data were analyzed from August to December 2022 using R statistical software version 4.1.2 (R Project for Statistical Computing).

## Results

Our sample included 54 older adults (33 [61%] female; mean [SD] age, 72 [7] years). Participants had a mean (SD) of 15 (3) years education and moderate depression severity (mean [SD] MADRS score, 25.3 [4.65]). Further details on the cohort are provided in the Table.

**Table.** Descriptive Statistics for Variables of Interest

Test name	Mean (SD)
Dementia Rating Scale^a^	139 (4.6)
Subjective social support^b^	16 (4.0)
Phonemic verbal fluency^c^	38 (15.0)
Semantic verbal fluency^d^	20 (5.3)
Inhibitory control^e^	−3.5 (8.4)
Set shifting^f^	74.4 (34.4)
Processing speed^g^	41.8 (14.1)
Memory^h^	7.1 (3.1)
Dysexecutive behavior^i^	43.2 (10.5)

^a^ Range, 124 to 144; higher scores indicate better global cognitive performance.

^b^ Assessed using the Duke Social Support Index (range, 7-21; higher scores indicate stronger perceived social support).

^c^ Assessed using the Controlled Oral Word Association Test (range, 0-67; higher scores indicate better phonemic verbal fluency performance).

^d^ Assessed using the Animal Naming Test (range, 10-34; higher scores indicate better semantic verbal fluency performance).

^e^ Assessed using the Stroop Interference test. Scores were calculated as ColorWord – ([Word × Color] / [Word + Color]) (range, −27.3 to 11.7; lower scores indicate greater difficulty with cognitive inhibition).

^f^ Assessed using the Trail Making Test part B minus part A (range, 24.8-166.4; higher scores indicate greater difficulty with set-shifting).

^g^ Assessed using the Trail Making Test part A (range, 19.5-77.0; higher scores indicate slower processing speed).

^h^ Assessed using the Hopkins Verbal Learning Test delayed recall (range, 0-12; higher scores indicate better delayed verbal memory performance).

^i^ Assessed using the Frontal Systems Behavior Scale (range, 21-65; higher scores indicate greater self-reported executive dysfunction).

Higher perceived social support was associated with higher FAS scores (B = 1.51; *P* = .005), controlling for age (B = −0.40; *P* = .17), sex (B = 5.76; *P* = .15), education (B = 3.00; *P* < .001), and MADRS scores (B = −0.20; *P* = .65) (F_5,45_ = 7.30; *P* < .001; *R*^2^ = 0.39) (Figure). In a reverse association model, we also found that higher FAS scores were associated with higher social support (B = 0.11; *P* = .005; F_5,45_ = 6.92; *R*^2^ = 0.37; *P* < .001)

**Figure.**
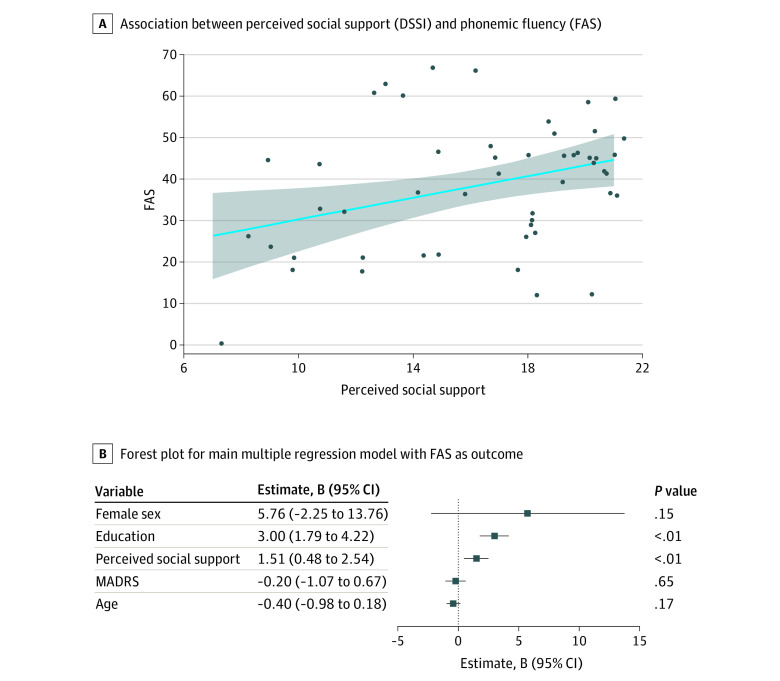
Association Between Perceived Social Support and Phonemic Verbal Fluency A, Shaded area represents the 95% confidence interval. Social support was measured with Duke Social Support Index (DSSI), and phonemic fluency (FAS) was measured with the Controlled Oral Word Association Test. In a follow-up sensitivity analysis removing 1 participant with an FAS score of 0, the findings remained unchanged, with higher social support associated with higher verbal fluency. MADRS indicates Montgomery-Åsberg Depression Rating Scale.

## Discussion

In this cross-sectional study among older adults with depression, higher perceived social support was associated with phonemic verbal fluency but not other executive processes or cognitive domains. Clinical and neuroimaging studies suggest that semantic fluency tasks rely more on lexical access and language abilities, while phonemic fluency places greater demands on executive processes.^6^ These results indicate that perceived social isolation may be selectively associated with executive processes that support initiation and persistence of behavior, underscoring the important role of social support in this population. Older adults with poorer cognitive performance may be less likely to feel supported by others and thus may benefit from interventions that increase social engagement. Study limitations include its cross-sectional design and our focus on specific dimensions of social support. Additionally, future studies could examine generalizability of these findings to more complex clinical populations, such as older adults with comorbid psychiatric conditions and those receiving antidepressant medications. Still, our findings could inform intervention development for older adults with depression that target social support to increase cognitive resilience.

## Notes

Supplement 1.
eMethods.

Supplement 2.
Data Sharing Statement
