# Supplementary material for: Perception of Social Support and Cognitive Performance in Older Adults With Depression
Source: JAMA Netw Open. 2023 Mar 21;6(3):e233978. doi: 10.1001/jamanetworkopen.2023.3978 (PMC10031397; doi:10.1001/jamanetworkopen.2023.3978)
Supplement: Supplement 1. — eMethods. [file jamanetwopen-e233978-s001.pdf]

Supplemental Online Content

Doreste-Mendez R, Oberlin LE, Ilieva I, Chen SZ, Gunning FM, Solomonov N. Perception of social support and cognitive performance in older adults with depression. *JAMA Netw Open*. 2023;6(3):e233978. doi:10.1001/jamanetworkopen.2023.3978

eMethods.

This supplemental material has been provided by the authors to give readers additional information about their work.

© 2023 Doreste-Mendez R et al. *JAMA Network Open.*

eMethods.

Protocol and Participants

The current study is a cross-sectional analysis focused on the baseline data from a larger clinical trial, the White Matter and Emotional and Cognitive Control in Late-Onset Depression study.[1] The clinical trial, including all assessments, were conducted at an outpatient geriatric psychiatry clinic within Weill Cornell Medicine from July 1, 2012, to July 31, 2019. Participants were recruited for a single-group, open-label escitalopram treatment trial (NCT01728194) through advertisements and clinical referrals. Details of the study can be found here.[2]

Our sample included 54 older adults aged 59 – 85 years who met criteria for Major Depressive Disorder without psychotic features according to the Diagnostic and Statistical Manual of Mental Disorders (Fourth Edition) (DSM-IV). Exclusion criteria included presence or history of any Axis I psychiatric disorder other than MDD or co-morbid generalized anxiety disorder; high suicide risk; history of electroconvulsive therapy; antidepressant treatment during the study period; acute or severe medical illness; MCI or dementia; history or presence of neurological disease. Of the 54 participants enrolled, 3 subjects did not complete the Duke Social Support Index (DSSI) and were excluded from analyses.

Measures

Perceived Social Support. The Duke Social Support Index (DSSI) is a self-report scale that measures multiple dimensions of social support. The DSSI has been found reliable and internally consistent in the aging population.[3] It includes subscales measuring instrumental support, size of social network, frequency of social interactions, and perceived social support – an individual‘s perception of the emotional support provided by others. In this study, we focused on the 6-item “Perceived Social Support” subscale, based on previous studies showing that this subscale is highly predictive of late-life depression severity, persistence over time, and

© 2023 Doreste-Mendez R et al. *JAMA Network Open.*

response to psychosocial interventions.[4–7] Items include questions such as: “Do you feel you have a definite role (place) in your family and among your friends?” and “Can you talk about your deepest problems with at least some of your family and friends?”. Items are scored on a 3-point Likert scale (1=Hardly Ever, 2=Some of the Time or 3=Most of the Time), and higher scores indicate stronger perceived social support. This subscale showed high internal consistency in our sample (Cronbach’s α=0.88).

Depression Severity. The Montgomery-Åsberg Depression Rating Scale (MADRS) is a widely used clinical scale that measures depression severity[8] and is commonly used in older adult populations. It includes 10 items that measure sadness, lassitude, inability to feel, pessimistic thoughts, inner tension, concentration difficulties, suicidal thoughts, and vegetative symptoms (sleep and appetite). Each of the items are scored on a scale of 0 – 6. The maximum total score is 60, with higher scores indicating greater depression severity.

Global Cognitive Functioning. The Dementia Rating Scale (DRS-2) is a measure of global cognition.[9] It includes multiple tasks that are grouped into 5 subscales: Attention, Initiation/Perseveration, Construction, Conceptualization, and Memory. The maximum total score is 144. Higher scores indicate better performance.

Phonemic Verbal Fluency. Phonemic Verbal Fluency was measured using the Controlled Oral Word Association Test (COWAT, also known as FAS). Participants were asked to orally generate as many words as they can that start with the letters F, A, S within a predetermined time frame (60-seconds per letter).[10] The outcome variable is the sum of the total number of words produced across all three letters, with higher scores reflecting better performance.[11]

Semantic Verbal Fluency. The Animal Naming Test (ANT) is a test of semantic fluency, in which the participant is asked to orally produce as many animal names as possible within sixty

seconds. The outcome variable is the total number of animal names produced, with higher scores reflecting better performance.

Cognitive Inhibition. The Stroop Interference index of the Stroop Color Word Test assesses inhibitory control. In the first two trials, participants are asked to read color words and name ink colors aloud as quickly as possible and the total number of correct items read in 45 seconds is recorded. In the third trial, color words are printed in an inconsistent color ink, and participants are asked to read aloud the ink color while ignoring the color name. Stroop interference score is calculated as ColorWord - [(Word x Color) / (Word + Color)].[12] Lower Stroop interference scores indicate greater difficulty with cognitive inhibition.

Memory. On the Hopkins Verbal Learning Test, participants are read a list of words several times, and asked to recall the words immediately and after a 20-minute delay. Total number of words recalled during the delayed trial served as the outcome variable, with higher scores indicating better performance.

Processing Speed. In Trail Making Test Part A (TMT-A), participants are shown circles on a piece of paper numbered from 1 – 25 and are asked to connect the circles in ascending numerical order as quickly as possible. Scores are recorded as time taken to complete the task. Higher time to completion is reflective of slower processing speed.

Set-Shifting. In Trail Making Test Part B (TMT-B), the participant is asked to draw a line connecting circles by alternating between numbers and letters in alphabetical and numerical order. Scores for TMT-B are recorded as time taken to complete the task. Set-shifting was measured by subtracting TMT-A completion time from TMT-B completion time (TMT B – A), with higher scores indicating greater difficulty with set-shifting.

Self-reported Executive Function. The Frontal Systems Behavior Scale (FrSBe) is a 46-item behavior rating scale designed to measure behavioral dysfunction associated with frontal

© 2023 Doreste-Mendez R et al. *JAMA Network Open.*

subcortical deficits. The scale provides a total score and includes three subscales: Apathy, Disinhibition, and Executive Dysfunction. For this study, we used the Executive Dysfunction subscale. Items in this subscale aim to measure issues with sustained attention, planning, sequencing, organization, problem-solving, self-monitoring, insight, and mental flexibility. Higher scores indicate greater self-reported executive dysfunction.

© 2023 Doreste-Mendez R et al. *JAMA Network Open.*

References

1. Gunning F. White matter and emotional and cognitive control in late-onset depression. ClinicalTrials.gov identifier: NCT01728194. Accessed November 20, 2022. https://clinicaltrials.gov/ct2/show/NCT01728194

2. Oberlin L, Victoria L, Ilieva I, Dunlop K, Hoptman M, Avari J, et al. Comparison of functional and structural neural network features in older adults with depression vs without apathy and association with response to escitalopram: Secondary analysis of a nonrandomized clinical trial. *JAMA Netw Open*. 2022;5:e2224142. doi:10.1001/jamanetworkopen.2022.24142

3. Koenig HG, Westlund RE, George LK, Hughes DC, Blazer DG, Hybels C. Abbreviating the Duke Social Support Index for use in chronically ill elderly individuals. *Psychosomatics*. 1993;34(1):61-69. doi:https://doi.org/10.1016/S0033-3182(93)71928-3

4. George LK, Blazer DG, Hughes DC, Fowler N. Social support and the outcome of major depression. *The British Journal of Psychiatry*. 1989;154(4):478-485. doi:DOI: 10.1192/bjp.154.4.478

5. Woods A, Solomonov N, Liles B, Guillod A, Kales HC, Sirey JA. Perceived social support and interpersonal functioning as predictors of treatment response among depressed older adults. *The American Journal of Geriatric Psychiatry*. 2021;29(8):843-852. doi:10.1016/J.JAGP.2020.12.021

6. Solomonov N, Lee J, Banerjee S, Flückiger C, Kanellopoulos D, Gunning FM, et al. Modifiable predictors of nonresponse to psychotherapies for late-life depression with executive dysfunction: a machine learning approach. *Mol Psychiatry*. 2021;26(9):5190-5198. doi:10.1038/s41380-020-0836-z

© 2023 Doreste-Mendez R et al. *JAMA Network Open.*

7. Oddone CG, Hybels CF, McQuoid DR, Steffens DC. Social support modifies the relationship between personality and depressive symptoms in older adults. *The American Journal of Geriatric Psychiatry*. 2011;19(2):123-131. doi:https://doi.org/10.1097/JGP.0b013e3181f7d89a

8. Montgomery SA, Asberg M. A new depression scale designed to be sensitive to change. *The British Journal of Psychiatry*. 1979;134(4):382-389. doi:10.1192/bjp.134.4.382

9. Mattis S. *Dementia Rating Scale: Professional Manual.* Psychological Assessment Resources, Inc.; 1988.

10. Strauss E, Sherman EMS, Spreen O. *A Compendium of Neuropsychological Tests: Administration, Norms, and Commentary.* Oxford University Press; 2006.

11. Tombaugh TN, Kozak J, Rees L. Normative data stratified by age and education for two measures of verbal fluency: FAS and Animal Naming. *Archives of Clinical Neuropsychology*. 1999;14(2):167-177. doi: https://doi.org/10.1016/S0887-6177(97)00095-4

12. Golden CJ. *Stroop Color and Word Test: A Manual for Clinical and Experimental Uses*. Stoelting Co.; 1978.

© 2023 Doreste-Mendez R et al. *JAMA Network Open.*
