# Supplementary material for: Perception of Social Support and Cognitive Performance in Older Adults With Depression
Source: JAMA Netw Open. 2023 Mar 21;6(3):e233978. doi: 10.1001/jamanetworkopen.2023.3978 (PMC10031397; doi:10.1001/jamanetworkopen.2023.3978)
Supplement: Supplement 2. — Data Sharing Statement [file jamanetwopen-e233978-s002.pdf]

Data Sharing Statement

Doreste-Mendez. Perception of Social Support and Cognitive Performance in Older Adults With Depression. *JAMA Netw Open*. Published March 21, 2023.
doi:10.1001/jamanetworkopen.2023.3978

Data
Data available: Yes
Data types: Other (please specify)
Additional Information: For data access, please reach out to the PI, Faith Gunning, to obtain project approval.
How to access data: fgd2002@med.cornell.edu
When available: With publication

Supporting Documents
Document types: None

Additional Information
Who can access the data: Researchers whose proposed use of the data has been approved.
Types of analyses: For a specified purpose.
Mechanisms of data availability: After approval of a proposal.
